# (*E*)-1-(4-Meth­oxy­anthracen-1-yl)-2-phenyl­diazene

**DOI:** 10.1107/S1600536811010932

**Published:** 2011-03-31

**Authors:** Aurelien Crochet, Katharina M. Fromm, Vanya Kurteva, Liudmil Antonov

**Affiliations:** a University of Fribourg, Department of Chemistry, Chemin du Musee 9, CH-1700 Fribourg, Switzerland; b Institute of Organic Chemistry, Bulgarian Academy of Sciences, Acad. G. Bonchev str., bdg 9, Sofia 1113, Bulgaria

## Abstract

The title compound, C_21_H_16_N_2_O, has an *E*-conformation about the diazene N=N bond. It is reasonably planar with the phenyl ring being inclined to the mean plane of the anthracene moiety [planar to within 0.077 (3) Å] by 6.43 (10)°. The crystal structure is stabilized by C—H⋯π and weak π–π inter­actions [centroid–centroid distances of 3.7192 (16) and 3.8382 (15) Å], leading to the formation of two-dimensional networks stacking along [001] and lying parallel to (110).

## Related literature

For background to sensing mol­ecules based on tautomeric switches, see: Nedeltcheva *et al.* (2009 ▶); Antonov *et al.* (2009 ▶, 2010 ▶). For investigations of the tautomerism of azodyes, see: Kelemen (1981 ▶). For the synthesis of the title compound, see: Nedeltcheva *et al.* (2010 ▶).
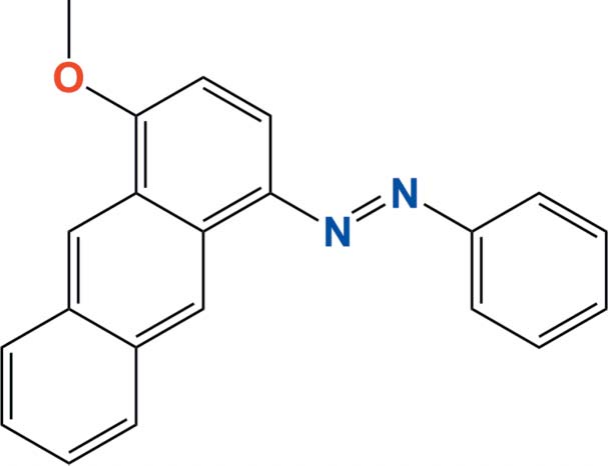


## Experimental

### 

#### Crystal data

C_21_H_16_N_2_O
*M* *_r_* = 312.36
Orthorhombic, 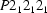

*a* = 6.3021 (3) Å
*b* = 9.0481 (4) Å
*c* = 27.3935 (17) Å
*V* = 1562.03 (14) Å^3^
*Z* = 4
Mo *K*α radiation
μ = 0.08 mm^−1^
*T* = 150 K
0.54 × 0.32 × 0.12 mm

#### Data collection

STOE IPDS 2T diffractometer
12181 measured reflections
2584 independent reflections
2096 reflections with *I* > 2σ(*I*)
*R*_int_ = 0.072

#### Refinement

*R*[*F* ^2^ > 2σ(*F* ^2^)] = 0.047
*wR*(*F* ^2^) = 0.086
*S* = 1.10
2584 reflections
218 parameters
H-atom parameters constrained
Δρ_max_ = 0.13 e Å^−3^
Δρ_min_ = −0.15 e Å^−3^

### 

Data collection: *X-AREA* (Stoe & Cie, 2009 ▶); cell refinement: *X-AREA*; data reduction: *X-RED32* (Stoe & Cie, 2009 ▶); program(s) used to solve structure: *SHELXS97* (Sheldrick, 2008 ▶); program(s) used to refine structure: *SHELXL97* (Sheldrick, 2008 ▶); molecular graphics: *PLATON* (Spek, 2009 ▶) and *Mercury* (Macrae *et al.*, 2006 ▶); software used to prepare material for publication: *SHELXL97*, *PLATON* and *publCIF* (Westrip, 2010 ▶).

## Supplementary Material

Crystal structure: contains datablocks I, global. DOI: 10.1107/S1600536811010932/nc2221sup1.cif

Structure factors: contains datablocks I. DOI: 10.1107/S1600536811010932/nc2221Isup2.hkl

Additional supplementary materials:  crystallographic information; 3D view; checkCIF report

## Figures and Tables

**Table 1:** C—H⋯π interactions (Å, °) *Cg*1, *Cg*2 and *Cg*3 are the centroids of the C1–C6, C7,C8,C17–C20 and C8–C10,C15–C17) rings, respectively.

C—H⋯*Cg*	C—H	H⋯*Cg*	C⋯*Cg*	C—H⋯*Cg*
C21—H21*A*⋯*Cg*1^i^	0.98	2.83	3.646 (4)	141
C12—H12⋯*Cg*2^ii^	0.95	2.80	3.681 (4)	154
C11—H11⋯*Cg*3^ii^	0.95	2.83	3.543 (3)	132

Symmetry codes: (i) 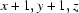; (ii) 
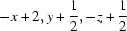
.

**Fig. 1.**
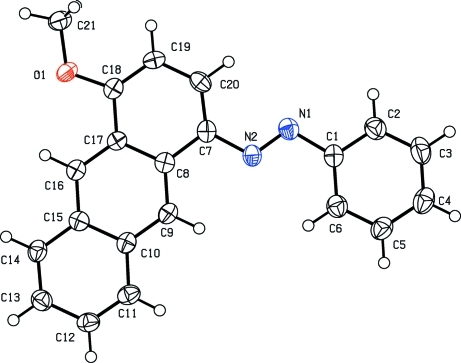
A view of the molecular structure of the title compound with labeling and displacement ellipsoids drawn at the 50% probability level.

**Fig. 2.**
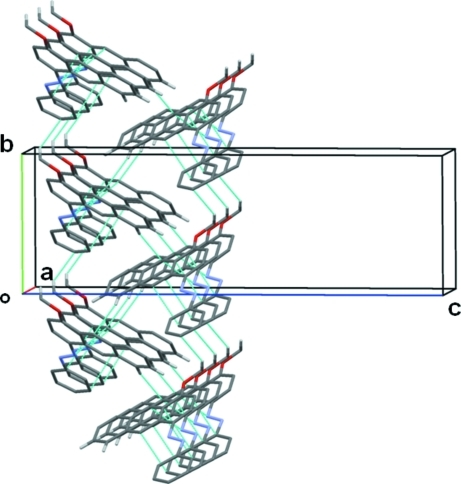
A view along the a-axis of the crystal structure of the title compound. The π···π and C—H···π and interactions are shown as dashed cyan lines [see Table 1 for details; H-atoms not involved in these interactions have been omitted for clarity].

## supplementary crystallographic information

### Comment

More than 90% of the existing commercial azodyes are tautomeric ones, which makes the investigation of their tautomerism of substantial practical interest (Kelemen, 1981). However, in most of the tautomeric dyes the tautomeric equilibrium cannot be shifted to the pure, end tautomeric forms. In such cases model compounds, possessing the characteristics of the corresponding end-structures, are usually applied. As a part of our interest in sensing molecules based on tautomeric switches (Nedeltcheva *et al.*, 2009, Antonov *et al.*, 2009, 2010), the equilibrium in 4-phenylazo-antracene-1-ol was studied in the gas phase by mass spectrometry, and in solution by flash photolysis (Nedeltcheva *et al.*, 2010). The corresponding *O*-methyl and *N*-methyl derivatives were used as model enol and keto tautomers, respectively, and the tautomeric constant was estimated. Herein, we report on the crystal structure of the title compound, the model enol analogue of 4-phenylazo-antracene-1-ol.

The molecular structure of the title molecule is shown in Fig. 1. The molecule, which has the *E*-conformation about the diazene N1═N2 bond, is relatively planar, with phenyl ring (C1—C6) being inclined to the mean plane of the anthracene moiety (C7—C20) by 6.43 (10) °.

In the crystal the molecules are linked by C—H···π interactions (Table 1). There are also weak π···π interactions involving the phenyl ring (C1—C6) with rings (C7,C8,C17—C20)^i^ and (C8—C10,C15—C17)^i^ [symmetry code (i) *x* - 1, *y* - 1, *z*]; the centroid-centroid distances are 3.7192 (16) and 3.8382 (15) Å, respectively. These interactions lead to the formation of two-dimensional sheet-like networks that stack along the *c* axis, lying parallel to the *ab*-plane (Fig. 2).

### Experimental

Simple methylation of (*E*)-4-(phenyldiazenyl)anthracen-1-ol in basic media gave an easy separable mixture of the title compound and the corresponding *N*-methyl derivative, with yields of 32% and 43%, respectively (Nedeltcheva *et al.*, 2010). Dark red block-like crystals of the title compound, suitable for X-ray diffraction analysis, were grown by slow diffusion of hexane into a chloroform solution of the title compound.

### Refinement

Because no heavy atoms are present the absolute structure and absolute configuration cannot be determined.Therefore, Friedel opposites were merged in the refinement. The C-bound H-atoms were included in calculated positions and treated as riding atoms: C—H = 0.95 and 0.98 Å for CH and CH_3_ H-atoms, respectively, with *U*_iso_(H) = k × *U*_eq_(C), where k = 1.5 for CH_3_ H-atoms, and k = 1.2 for all other H-atoms.

### Crystal data

**Table:**

C_21_H_16_N_2_O	*F*(000) = 656
*M**_r_* = 312.36	*D*_x_ = 1.328 Mg m^−^^3^
Orthorhombic, *P*2_1_2_1_2_1_	Mo *K*α radiation, λ = 0.71073 Å
Hall symbol: P 2ac 2ab	Cell parameters from 8137 reflections
*a* = 6.3021 (3) Å	θ = 1.5–25.1°
*b* = 9.0481 (4) Å	µ = 0.08 mm^−^^1^
*c* = 27.3935 (17) Å	*T* = 150 K
*V* = 1562.03 (14) Å^3^	Block, red
*Z* = 4	0.54 × 0.32 × 0.12 mm

### Data collection

**Table:**

STOE IPDS 2T diffractometer	2096 reflections with *I* > 2σ(*I*)
Radiation source: fine-focus sealed tube	*R*_int_ = 0.072
graphite	θ_max_ = 24.6°, θ_min_ = 1.5°
Detector resolution: 6.67 pixels mm^-1^	*h* = −6→7
rotation method scans	*k* = −9→10
12181 measured reflections	*l* = −32→32
2584 independent reflections	

### Refinement

**Table:**

Refinement on *F*^2^	Primary atom site location: structure-invariant direct methods
Least-squares matrix: full	Secondary atom site location: difference Fourier map
*R*[*F*^2^ > 2σ(*F*^2^)] = 0.047	Hydrogen site location: inferred from neighbouring sites
*wR*(*F*^2^) = 0.086	H-atom parameters constrained
*S* = 1.10	*w* = 1/[σ^2^(*F*_o_^2^) + (0.0342*P*)^2^ + 0.1352*P*] where *P* = (*F*_o_^2^ + 2*F*_c_^2^)/3
2584 reflections	(Δ/σ)_max_ < 0.001
218 parameters	Δρ_max_ = 0.13 e Å^−^^3^
0 restraints	Δρ_min_ = −0.15 e Å^−^^3^

### Special details

**Table:**

Geometry. All s.u.'s (except the s.u. in the dihedral angle between two l.s. planes) are estimated using the full covariance matrix. The cell s.u.'s are taken into account individually in the estimation of s.u.'s in distances, angles and torsion angles; correlations between s.u.'s in cell parameters are only used when they are defined by crystal symmetry. An approximate (isotropic) treatment of cell s.u.'s is used for estimating s.u.'s involving l.s. planes.
Refinement. Refinement of *F*^2^ against ALL reflections. The weighted *R*-factor *wR* and goodness of fit *S* are based on *F*^2^, conventional *R*-factors *R* are based on *F*, with *F* set to zero for negative *F*^2^. The threshold expression of *F*^2^ > 2σ(*F*^2^) is used only for calculating *R*-factors(gt) *etc*. and is not relevant to the choice of reflections for refinement. *R*-factors based on *F*^2^ are statistically about twice as large as those based on *F*, and *R*- factors based on ALL data will be even larger.

### Fractional atomic coordinates and isotropic or equivalent isotropic displacement parameters (Å^2^)

**Table:**

	*x*	*y*	*z*	*U*_iso_*/*U*_eq_	
C1	0.3714 (4)	−0.0729 (3)	0.41632 (8)	0.0295 (6)	
C2	0.2333 (4)	−0.0908 (3)	0.45491 (10)	0.0390 (7)	
H2	0.2609	−0.0433	0.4852	0.047*	
C3	0.0531 (5)	−0.1787 (3)	0.44947 (11)	0.0459 (8)	
H3	−0.0422	−0.1911	0.4760	0.055*	
C4	0.0136 (5)	−0.2471 (3)	0.40569 (10)	0.0428 (8)	
H4	−0.1105	−0.3056	0.4018	0.051*	
C5	0.1533 (5)	−0.2314 (3)	0.36735 (11)	0.0410 (7)	
H5	0.1260	−0.2802	0.3373	0.049*	
C6	0.3333 (4)	−0.1448 (3)	0.37242 (9)	0.0343 (6)	
H6	0.4300	−0.1348	0.3460	0.041*	
C7	0.8346 (4)	0.1413 (3)	0.39361 (8)	0.0279 (6)	
C8	0.9818 (4)	0.1429 (3)	0.35326 (8)	0.0278 (6)	
C9	0.9493 (4)	0.0641 (3)	0.31029 (8)	0.0308 (6)	
H9	0.8233	0.0075	0.3067	0.037*	
C10	1.0975 (4)	0.0660 (3)	0.27227 (8)	0.0287 (6)	
C11	1.0656 (5)	−0.0128 (3)	0.22788 (8)	0.0335 (6)	
H11	0.9396	−0.0689	0.2235	0.040*	
C12	1.2126 (5)	−0.0087 (3)	0.19165 (8)	0.0352 (7)	
H12	1.1875	−0.0608	0.1621	0.042*	
C13	1.4030 (4)	0.0725 (3)	0.19757 (9)	0.0355 (7)	
H13	1.5051	0.0736	0.1721	0.043*	
C14	1.4408 (4)	0.1487 (3)	0.23939 (8)	0.0328 (6)	
H14	1.5688	0.2032	0.2428	0.039*	
C15	1.2913 (4)	0.1479 (3)	0.27795 (8)	0.0280 (6)	
C16	1.3235 (4)	0.2270 (3)	0.32118 (8)	0.0290 (6)	
H16	1.4516	0.2808	0.3253	0.035*	
C17	1.1728 (4)	0.2290 (3)	0.35837 (8)	0.0258 (6)	
C18	1.2018 (4)	0.3159 (3)	0.40158 (8)	0.0284 (6)	
C19	1.0512 (4)	0.3180 (3)	0.43775 (8)	0.0302 (6)	
H19	1.0691	0.3792	0.4656	0.036*	
C20	0.8696 (4)	0.2281 (3)	0.43313 (8)	0.0322 (7)	
H20	0.7677	0.2285	0.4587	0.039*	
C21	1.4285 (5)	0.4842 (3)	0.44324 (8)	0.0410 (7)	
H21A	1.3165	0.5585	0.4470	0.061*	
H21B	1.5656	0.5337	0.4390	0.061*	
H21C	1.4333	0.4217	0.4724	0.061*	
N1	0.5491 (4)	0.0227 (3)	0.42517 (7)	0.0329 (5)	
N2	0.6573 (3)	0.0465 (2)	0.38713 (7)	0.0301 (5)	
O1	1.3851 (3)	0.3951 (2)	0.40156 (6)	0.0347 (5)	

### Atomic displacement parameters (Å^2^)

**Table:**

	*U*^11^	*U*^22^	*U*^33^	*U*^12^	*U*^13^	*U*^23^
C1	0.0235 (15)	0.0275 (16)	0.0375 (13)	0.0038 (13)	0.0023 (11)	0.0052 (11)
C2	0.0385 (18)	0.0367 (19)	0.0418 (14)	−0.0030 (14)	0.0075 (13)	−0.0038 (13)
C3	0.0361 (18)	0.044 (2)	0.0573 (17)	−0.0027 (16)	0.0182 (15)	−0.0014 (15)
C4	0.0335 (19)	0.0329 (18)	0.0620 (19)	0.0001 (14)	0.0059 (15)	−0.0030 (14)
C5	0.0377 (18)	0.0352 (18)	0.0500 (16)	−0.0040 (15)	−0.0016 (14)	−0.0009 (13)
C6	0.0330 (16)	0.0341 (16)	0.0359 (13)	0.0017 (14)	0.0032 (12)	0.0043 (12)
C7	0.0220 (15)	0.0298 (16)	0.0319 (12)	0.0031 (13)	−0.0034 (11)	0.0060 (11)
C8	0.0294 (15)	0.0280 (15)	0.0260 (12)	0.0022 (13)	−0.0010 (11)	0.0037 (10)
C9	0.0253 (14)	0.0341 (16)	0.0330 (13)	−0.0021 (13)	−0.0042 (12)	0.0013 (11)
C10	0.0272 (15)	0.0274 (15)	0.0314 (12)	0.0005 (13)	−0.0018 (11)	0.0003 (11)
C11	0.0357 (16)	0.0324 (16)	0.0322 (13)	−0.0038 (13)	−0.0045 (12)	−0.0007 (11)
C12	0.0410 (17)	0.0361 (17)	0.0285 (12)	−0.0008 (14)	−0.0017 (12)	−0.0026 (12)
C13	0.0365 (17)	0.0337 (17)	0.0363 (14)	0.0002 (15)	0.0047 (12)	−0.0013 (12)
C14	0.0291 (16)	0.0315 (16)	0.0378 (13)	−0.0024 (13)	0.0033 (12)	0.0005 (11)
C15	0.0314 (15)	0.0253 (15)	0.0273 (12)	0.0023 (12)	−0.0010 (12)	0.0025 (11)
C16	0.0247 (15)	0.0283 (16)	0.0341 (13)	0.0011 (12)	−0.0026 (12)	0.0021 (10)
C17	0.0254 (15)	0.0260 (15)	0.0259 (12)	0.0007 (12)	−0.0028 (11)	0.0013 (10)
C18	0.0265 (15)	0.0275 (16)	0.0312 (12)	−0.0005 (12)	−0.0048 (12)	0.0023 (11)
C19	0.0316 (16)	0.0314 (16)	0.0276 (12)	0.0030 (13)	−0.0025 (12)	−0.0038 (11)
C20	0.0312 (16)	0.0368 (17)	0.0285 (13)	0.0060 (14)	0.0018 (12)	0.0003 (11)
C21	0.0461 (18)	0.0429 (18)	0.0340 (13)	−0.0091 (15)	−0.0066 (13)	−0.0066 (12)
N1	0.0315 (13)	0.0331 (13)	0.0342 (11)	0.0031 (11)	0.0053 (10)	0.0021 (9)
N2	0.0246 (12)	0.0333 (14)	0.0325 (11)	0.0033 (11)	0.0005 (10)	0.0042 (9)
O1	0.0350 (12)	0.0380 (11)	0.0311 (9)	−0.0086 (9)	−0.0036 (8)	−0.0065 (8)

### Geometric parameters (Å, °)

**Table:**

C1—C2	1.379 (3)	C11—H11	0.9500
C1—C6	1.389 (3)	C12—C13	1.416 (4)
C1—N1	1.435 (3)	C12—H12	0.9500
C2—C3	1.394 (4)	C13—C14	1.358 (3)
C2—H2	0.9500	C13—H13	0.9500
C3—C4	1.373 (4)	C14—C15	1.415 (3)
C3—H3	0.9500	C14—H14	0.9500
C4—C5	1.378 (4)	C15—C16	1.398 (3)
C4—H4	0.9500	C16—C17	1.393 (3)
C5—C6	1.386 (4)	C16—H16	0.9500
C5—H5	0.9500	C17—C18	1.433 (3)
C6—H6	0.9500	C18—O1	1.359 (3)
C7—C20	1.356 (3)	C18—C19	1.372 (3)
C7—N2	1.420 (3)	C19—C20	1.409 (4)
C7—C8	1.443 (3)	C19—H19	0.9500
C8—C9	1.391 (3)	C20—H20	0.9500
C8—C17	1.441 (4)	C21—O1	1.424 (3)
C9—C10	1.399 (3)	C21—H21A	0.9800
C9—H9	0.9500	C21—H21B	0.9800
C10—C11	1.423 (3)	C21—H21C	0.9800
C10—C15	1.437 (4)	N1—N2	1.264 (3)
C11—C12	1.358 (4)		
			
C2—C1—C6	120.0 (3)	C13—C12—H12	119.7
C2—C1—N1	115.7 (2)	C14—C13—C12	120.6 (2)
C6—C1—N1	124.3 (2)	C14—C13—H13	119.7
C1—C2—C3	120.0 (3)	C12—C13—H13	119.7
C1—C2—H2	120.0	C13—C14—C15	120.7 (3)
C3—C2—H2	120.0	C13—C14—H14	119.7
C4—C3—C2	119.9 (3)	C15—C14—H14	119.7
C4—C3—H3	120.1	C16—C15—C14	122.2 (2)
C2—C3—H3	120.1	C16—C15—C10	118.6 (2)
C3—C4—C5	120.2 (3)	C14—C15—C10	119.2 (2)
C3—C4—H4	119.9	C17—C16—C15	121.8 (2)
C5—C4—H4	119.9	C17—C16—H16	119.1
C4—C5—C6	120.3 (3)	C15—C16—H16	119.1
C4—C5—H5	119.8	C16—C17—C18	121.6 (2)
C6—C5—H5	119.8	C16—C17—C8	119.4 (2)
C5—C6—C1	119.6 (3)	C18—C17—C8	118.9 (2)
C5—C6—H6	120.2	O1—C18—C19	125.5 (2)
C1—C6—H6	120.2	O1—C18—C17	113.4 (2)
C20—C7—N2	125.3 (2)	C19—C18—C17	121.0 (2)
C20—C7—C8	120.1 (2)	C18—C19—C20	119.3 (2)
N2—C7—C8	114.6 (2)	C18—C19—H19	120.4
C9—C8—C17	118.8 (2)	C20—C19—H19	120.4
C9—C8—C7	123.2 (2)	C7—C20—C19	122.5 (2)
C17—C8—C7	117.9 (2)	C7—C20—H20	118.7
C8—C9—C10	121.7 (2)	C19—C20—H20	118.7
C8—C9—H9	119.2	O1—C21—H21A	109.5
C10—C9—H9	119.2	O1—C21—H21B	109.5
C9—C10—C11	122.4 (2)	H21A—C21—H21B	109.5
C9—C10—C15	119.5 (2)	O1—C21—H21C	109.5
C11—C10—C15	118.1 (2)	H21A—C21—H21C	109.5
C12—C11—C10	121.0 (3)	H21B—C21—H21C	109.5
C12—C11—H11	119.5	N2—N1—C1	112.59 (19)
C10—C11—H11	119.5	N1—N2—C7	115.1 (2)
C11—C12—C13	120.5 (2)	C18—O1—C21	117.5 (2)
C11—C12—H12	119.7		
			
C6—C1—C2—C3	−1.3 (4)	C11—C10—C15—C14	0.7 (4)
N1—C1—C2—C3	178.4 (3)	C14—C15—C16—C17	178.1 (2)
C1—C2—C3—C4	0.0 (4)	C10—C15—C16—C17	−0.4 (4)
C2—C3—C4—C5	1.1 (4)	C15—C16—C17—C18	−176.9 (2)
C3—C4—C5—C6	−0.9 (4)	C15—C16—C17—C8	2.5 (4)
C4—C5—C6—C1	−0.5 (4)	C9—C8—C17—C16	−2.4 (3)
C2—C1—C6—C5	1.6 (4)	C7—C8—C17—C16	176.7 (2)
N1—C1—C6—C5	−178.2 (3)	C9—C8—C17—C18	177.0 (2)
C20—C7—C8—C9	−175.8 (3)	C7—C8—C17—C18	−3.9 (3)
N2—C7—C8—C9	3.0 (4)	C16—C17—C18—O1	1.0 (3)
C20—C7—C8—C17	5.1 (4)	C8—C17—C18—O1	−178.4 (2)
N2—C7—C8—C17	−176.1 (2)	C16—C17—C18—C19	179.4 (2)
C17—C8—C9—C10	0.3 (4)	C8—C17—C18—C19	0.0 (4)
C7—C8—C9—C10	−178.8 (2)	O1—C18—C19—C20	−179.1 (2)
C8—C9—C10—C11	−179.3 (3)	C17—C18—C19—C20	2.7 (4)
C8—C9—C10—C15	1.7 (4)	N2—C7—C20—C19	178.8 (2)
C9—C10—C11—C12	−180.0 (3)	C8—C7—C20—C19	−2.6 (4)
C15—C10—C11—C12	−1.0 (4)	C18—C19—C20—C7	−1.5 (4)
C10—C11—C12—C13	0.9 (4)	C2—C1—N1—N2	−173.3 (2)
C11—C12—C13—C14	−0.6 (4)	C6—C1—N1—N2	6.5 (3)
C12—C13—C14—C15	0.4 (4)	C1—N1—N2—C7	179.6 (2)
C13—C14—C15—C16	−179.0 (3)	C20—C7—N2—N1	−13.9 (4)
C13—C14—C15—C10	−0.4 (4)	C8—C7—N2—N1	167.4 (2)
C9—C10—C15—C16	−1.7 (4)	C19—C18—O1—C21	2.2 (4)
C11—C10—C15—C16	179.3 (2)	C17—C18—O1—C21	−179.5 (2)
C9—C10—C15—C14	179.7 (2)		

### Hydrogen-bond geometry (Å, °)

**Table:**

Cg1, Cg2 and Cg3 are the centroids of the C1–C6, C7,C8,C17–C20 and C8–C10,C15–C17) rings, respectively.

**Table:**

*D*—H···*A*	*D*—H	H···*A*	*D*···*A*	*D*—H···*A*
C21—H21A···Cg1^i^	0.98	2.83	3.646 (4)	141
C12—H12···Cg2^ii^	0.95	2.80	3.681 (4)	154
C11—H11···Cg3^ii^	0.95	2.83	3.543 (3)	132

Symmetry codes: (i) *x*+1, *y*+1, *z*; (ii) −*x*+2, *y*+1/2, −*z*+1/2.
